# Evaluation of Light Physical Activity Measured by Accelerometry and Mobility Disability During a 6-Year Follow-up in Older Women

**DOI:** 10.1001/jamanetworkopen.2021.0005

**Published:** 2021-02-23

**Authors:** Nicole L. Glass, John Bellettiere, Purva Jain, Michael J. LaMonte, Andrea Z. LaCroix

**Affiliations:** 1 Herbert Wertheim School of Public Health and Human Longevity Science, University of California San Diego, La Jolla; 2 Department of Epidemiology and Environmental Health, School of Public Health and Health Professions, State University of New York at Buffalo, Buffalo

## Abstract

**Importance:**

Almost 1 in 4 women older than 65 years is unable to walk 2 to 3 blocks, and mobility disability is a key factor associated with loss of independence. Lack of moderate to vigorous–intensity physical activity is associated with mobility disability, but whether lighter physical activity is associated with mobility disability is unknown.

**Objective:**

To determine the association of light-intensity physical activity and incident mobility disability among older women.

**Design, Setting, and Participants:**

This prospective cohort study included women enrolled in the Objectively Measured Physical Activity and Cardiovascular Health study, an ancillary study of the Women’s Health Initiative, between March 2012 and April 2014, with follow-up through March 31, 2018. The Women’s Health Initiative was a population-based, multisite study that recruited from 40 clinical sites across the US. Participants in the present analysis included 5735 of 7058 ambulatory, community-dwelling women aged 63 years and older who returned an accelerometer with usable data, were free of mobility disability, and had follow-up data on mobility status. Data were analyzed from August 2018 to May 2019.

**Exposures:**

Light-intensity physical activity, defined as movement requiring energy expenditure between 1.6 and 2.9 metabolic equivalents, captured using an accelerometer over 7 days.

**Main Outcomes and Measures:**

Incident mobility disability, defined as the first self-reported inability to walk 1 block or up a flight of stairs at annual follow-up, and persistent incident mobility disability, defined as incident mobility loss that persisted through the end of follow-up.

**Results:**

A total of 5735 participants were included for primary analysis of all incident mobility disability (mean [SD] age, 78.5 [6.6] years [range, 63-97 years]; 2811 [49.0%] White participants). Compared with women in the lowest quartile of light-intensity physical activity, lower risk of incident mobility disability was observed in quartile 2 (multivariable hazard ratio [HR], 0.78; 95% CI, 0.67-0.90), quartile 3 (HR, 0.60; 95% CI, 0.51-0.71), and quartile 4 (HR, 0.60; 95% CI, 0.51-0.71) (*P* < .001). This beneficial association was stronger for persistent mobility disability in quartile 2 (multivariable HR, 0.72; 95% CI, 0.60-0.85), quartile 3 (HR, 0.55; 95% CI, 0.46-0.67), and quartile 4 (HR, 0.52; 95% CI, 0.42-0.63) (*P* < .001). Stratified analyses showed the association was stronger among women with a body mass index of less than 30.0 (HR, 0.73; 95% CI, 0.66-0.82) compared with women with a body mass index of 30.0 or higher (HR, 0.91; 95% CI; 0.79-1.04; *P* = .04 for interaction).

**Conclusions and Relevance:**

In this cohort study, increased time spent in light-intensity physical activity was associated with reduced incident mobility disability. These findings support placing greater emphasis on promoting light-intensity physical activity for preserving mobility in later life.

## Key Points

**Question:**

Is there an association between light-intensity physical activity and incident mobility disability among older women?

**Findings:**

In this cohort study of 5735 postmenopausal, community-dwelling women without mobility limitation, the risk of incident mobility disability over 6 years of follow-up was 22%, 40%, and 40% lower for women in the second, third, and fourth quartiles of daily mean light-intensity physical activity, respectively, compared with the lowest quartile.

**Meaning:**

These results suggest that recommendations to increase light-intensity physical activity have the potential to improve prospects for preserving mobility among older women.

## Teaser

This cohort study evaluates data from 40 clinical sites across the US to assess the association of light-intensity physical activity and incident mobility disability among older women.

## Introduction

Adults older than 65 years in the US are projected to reach 78 million people by 2035, outnumbering children under 18 years for the first time in US history.^1^ The aging of the US population is, in part, driven by increasing life expectancy, with women of all races/ethnicities holding a longevity advantage of almost 5 years over men.^2,3^ Even after adjusting for women’s longevity advantage, women have higher rates of morbidities, disabilities, and health care costs than men.^4,5,6^ Mobility disability, defined by the US Centers for Disease Control and Prevention as serious difficulty walking or climbing stairs, is the leading form of disability in the US and a fundamental quality-of-life issue affecting 27% of adults older than 65 years.^3,5,7^ Higher rates of mobility disability are observed among older women, with 24% unable to walk 2 to 3 blocks compared with 14% of older men.^3^ Older adults with mobility disability on average experience more hospitalizations and spend more than $2700 more in annual health care costs compared with those without mobility disability.^8^

Nearly all the evidence to date regarding physical activity (PA) and mobility, including that in the 2018 PA guidelines, has focused on higher-intensity PA.^9^ The Lifestyle Interventions and Independence for Elders (LIFE) study, a randomized clinical trial among older adults with existing physical limitations, showed that a program of structured moderate-intensity PA increased lower extremity physical functioning and reduced incident mobility disability,^10^ providing evidence that higher-intensity PA can prevent mobility disability. Whether light-intensity physical activity (LPA), such as casual walking or engaging in most daily life activities, reduces the risk of mobility disability is unknown and is among the major evidence gaps in the fields of aging and PA.^9^ Recent studies have found an association between accelerometer-measured LPA and decreased mortality and incident cardiovascular disease.^11,12^ Studying LPA is particularly important for older adults, because moderate to vigorous–intensity PA (MVPA) is increasingly difficult to perform, and engagement in MVPA declines with increasing age.^13^ A main reason for the lack of evidence related to LPA is because it is poorly measured by self-reporting, with a correlation to accelerometer-measured LPA of between 0.04 and 0.16.^14^ To our knowledge, this is the first study of accelerometer-measured LPA and incident mobility disability among community-dwelling older adults with intact mobility. The objective of this study was to investigate the association of accelerometer-measured LPA and incident mobility disability over 6 years of follow-up in a large diverse cohort of older women.

## Methods

### Study Participants

Between 1993 and 1998 the Women’s Health Initiative (WHI) enrolled postmenopausal women, aged 50 to 79 years, from 40 clinical sites across the US.^15,16^ The present cohort study includes women who participated in the Objectively Measured Physical Activity and Cardiovascular Health (OPACH) study, a longitudinal cohort and ancillary study of the WHI. In the OPACH study, consent was obtained from 7058 ambulatory community-dwelling women aged 63 years or older from March 2012 to April 2014.^17^ The OPACH study provided an ActiGraph GT3X+ accelerometer (ActiGraph Corp) to be worn over the right hip 24 hours per day for 7 days, except when the device could be submerged in water. Participants concurrently kept logs of their in-bed and out-of-bed times each night.

Our primary analytic sample of all incident mobility disability included 5735 women from the original OPACH cohort (eFigure 1 in the Supplement). Participants were excluded for death before receipt of an accelerometer (n = 10), not returning an accelerometer (n = 327) or returning the accelerometer with no usable data (n = 232), no days with 10 or more waking hours of wear time (n = 107), mobility disability at baseline (n = 602), or lack of follow-up for mobility status (n = 45). In the secondary analyses examining only persistent mobility disability, 418 women with incident mobility disability and reported recovery were excluded to provide a population at risk throughout follow-up (n = 5317). The protocol for the OPACH study was approved by the Fred Hutchinson Cancer Research Center institutional review board, and all women provided informed consent either in writing or orally by telephone. This report followed the Strengthening the Reporting of Observational Studies in Epidemiology (STROBE) reporting guideline.

### Mobility Disability

The definition of mobility was consistent with previously published studies in older adults as self-reported ability to walk 1 block and up a flight of stairs during a typical day.^18^ Mobility status was collected annually via mail or telephone from the OPACH study baseline through March 31, 2018. Incident mobility disability was defined as the first report of inability to walk 1 block or up a flight of stairs. Persistent mobility disability, defined as incident mobility loss persisting through the end of follow-up, was a secondary outcome, representing a more severe form of mobility loss with a stronger association with mortality.^19^

### PA Measures

Accelerometer data, measured between March 2012 and April 2014, using the ActiGraph GT3X+, were originally collected at 30 Hz and then integrated to 15-second epochs using the normal-frequency filter within ActiLife version 6 software (ActiGraph Corp). Accelerometer nonwear periods were identified and removed using the Choi algorithm.^17,20^ Sleep time was removed using reported in-bed and out-of-bed times from sleep logs. Missing bedtimes were imputed using participant-specific mean times or, if all data were missing, the OPACH population mean.

LPA includes all movement requiring energy expenditure between 1.6 and 2.9 metabolic equivalents. Examples of LPA include washing and drying dishes, gardening, and walking at a pace of about 1.5 mph, such as while shopping. Accelerometers were calibrated specifically for older women in a separate laboratory study of 200 women from the same OPACH population.^21^ LPA was computed as the mean number of hours per day with sufficient movement that the vector magnitude counts per 15-second epoch were between 19 and 518.^21^ MVPA, defined as activity requiring 3.0 or more metabolic equivalents, was measured as the mean number of minutes per day with vector magnitude counts per 15-second epoch of at least 519. As recommended, PA measures were computed using data from days with 10 or more hours of awake wear time.^22^ Mean hours per day of LPA were categorized into quartiles based on the distribution in the analytical sample (first quartile [Q1], 0.6 to ≤4.0 mean h/d of LPA; second quartile [Q2], >4.0 to ≤4.8 mean h/d of LPA; third quartile [Q3], >4.8 to ≤5.6 mean h/d of LPA; and fourth quartile [Q4], >5.6 to 10.4 mean h/d of LPA).

### Covariates

Potential confounding and effect-modifying variables were identified a priori based on published literature. All covariates were captured at OPACH baseline or by the WHI questionnaire completed closest before the OPACH baseline and included age, race/ethnicity (White, Black, or Hispanic/Latina), education (≤high school, some college, or college graduate), current smoking status, alcohol use (nondrinker, <1 drink/week, ≥1 drink/week, or unknown), continuous MVPA, body mass index (BMI, calculated as weight in kilograms divided by height in meters squared), multimorbidities (0, 1-2, or ≥3 from the sum of cancer, cerebrovascular disease, cognitive impairment, sensory impairment, cardiovascular disease, chronic obstructive pulmonary disease, diabetes, frequent falls, urinary incontinence, depression, and osteoarthritis), self-rated health (excellent/very good, good, or fair/poor), and short physical performance battery (SPPB) score (0-9 [low function] or 10-12 [high function]).^23^

### Statistical Analysis

To address differences in sleep time or nonwear time, PA measures were adjusted for awake wear time using the residuals method.^24^ Descriptive statistics were computed by quartile of LPA. Differences in characteristics across LPA categories were tested for statistical significance using analysis of variance for continuous variables and Pearson χ^2^ for categorical variables.

Hazard ratios (HRs) and 95% CIs for incident mobility disability (all and persistent) were determined using Cox proportional hazards regression models by quartiles of LPA in successively adjusted models. Person-years for each participant were calculated from OPACH baseline (first day of accelerometer wear) until first reported incident mobility disability, end of follow-up, or death, whichever occurred first. The proportional hazards assumption was assessed based on graphical inspection of survival curves. The minimally adjusted model (model 1) adjusted for age, race/ethnicity, and education. A confounder-adjusted model (model 2) further adjusted for health factors and behaviors associated with LPA and mobility disability (smoking, alcohol use, multimorbidities, and self-rated health). Model 3 adjusted for all model 2 covariates and MVPA.

To evaluate the consistency of associations among older women at higher vs lower risk of incident mobility loss, we conducted stratified analyses using the confounder-adjusted Cox regression model (model 2) by younger vs older age (median, 79 years; range, 63-97 years), race/ethnicity (White, Black, and Hispanic/Latina), high vs low MVPA (median, 45 min/d; range, 0-351 min/day), BMI (<30.0 vs ≥30.0), and high vs low SPPB score (0-9 vs ≥10). Interaction terms were added to the multivariable-adjusted Cox regression model to test for potential effect modification at α = .10, with age, MVPA, BMI, and SPPB score included as continuous terms.

The dose-response association of continuous LPA with incident mobility disability was then visualized using plots of the confounder-adjusted Cox regression model (model 2) and MVPA model (model 3) with restricted cubic-spline functions with 3 knots. Linearity of the dose-response association was tested using Wald tests. For reporting, HRs were calculated at 4, 5, 6, and 7 mean hours of LPA per day relative to the tenth percentile of LPA (3.3 hours per day).

All CIs were calculated at 95% and with an α of .05 unless otherwise specified. All analyses were conducted from August 2018 to May 2019, using RStudio version 1.1.463 for Mac (R Foundation) with the survival (survival analysis) and rms (regression modeling strategies) packages. All *P* values were 2-sided, and *P* < .05 was considered significant.

## Results

A total of 5735 participants were included for primary analysis of all incident mobility disability (mean [SD] age, 78.5 [6.6] years [range, 63-97 years]; 2811 [49.0%] White participants). A total of 2407 participants (41.9%) were college graduates, 3286 (57.3%) had less than a college degree, and 3068 (53.5%) had excellent or very good health (Table 1). Mean (SD) daily time spent in LPA was 4.8 (1.2) hours (range, 0.6-10.4 hours). Women with higher mean daily LPA were younger (mean [SD] age, 77.3 [6.5] years in Q4 vs 79.7 [6.6] years in Q1), were less likely to smoke (31 current smokers [2.2%] in Q4 vs 55 [3.8%] in Q1), were more likely to consume alcohol (436 [30.4%] consuming ≥1 drink/wk in Q4 vs 339 [23.6%] in Q1), had lower mean (SD) BMI (26.2 [5.0] in Q4 vs 29.8 [6.0] in Q1), had fewer chronic conditions (312 [21.9%] with no multimorbidities in Q4 vs 244 [17.1%] in Q1), had better self-rated health (815 [57.1%] reporting excellent or very good health in Q4 vs 730 [51.0%] in Q1), were more likely to have a high-function SPPB score (539 [42.6%] in Q4 vs 349 [29.2%] in Q1), and had a higher mean (SD) daily time in MVPA (66.8 [35.7] min/d in Q4 vs 36.1 [26.1] min/d in Q1).

**Table 1.** Baseline Characteristics by Quartile of Time Spent in Light-Intensity Physical Activity, WHI OPACH Study (n = 5735)

Characteristic	Participant by quartile of physical activity, NO. (%)^a^^,^^b^				*P* value
	1 (n = 1434)	2 (n = 1434)	3 (n = 1434)	4 (n = 1433)	
Light-intensity physical activity, mean (SD), h/d^a^	3.3 (0.5)	4.4 (0.2)	5.2 (0.2)	6.4 (0.6)	NA
Age, mean (SD), y	79.7 (6.6)	78.7 (6.7)	78.1 (6.5)	77.3 (6.5)	<.001
Race/ethnicity					
White	876 (61.1)	729 (50.8)	645 (45.0)	561 (39.2)	<.001
Black	388 (27.1)	477 (33.3)	517 (36.1)	542 (37.8)	
Hispanic/Latina	170 (11.9)	228 (15.9)	272 (19.0)	330 (23.0)	
BMI, mean (SD)	29.8 (6.0)	28.2 (5.4)	27.3 (5.2)	26.2 (5.0)	<.001
Highest education					
High school or less	248 (17.4)	278 (19.6)	279 (19.7)	301 (21.0)	.14
Some college	570 (40.1)	563 (39.6)	523 (36.9)	524 (36.6)	
College graduate	605 (42.5)	580 (40.8)	616 (43.4)	606 (42.4)	
Current smoker	55 (3.8)	38 (2.7)	23 (1.6)	31 (2.2)	.001
Alcohol consumption					
Nondrinker	508 (35.4)	469 (32.7)	455 (31.7)	455 (31.8)	.003
<1 drink per wk	452 (31.5)	475 (33.1)	460 (32.1)	429 (29.9)	
≥1 drink per wk	339 (23.6)	369 (25.7)	412 (28.7)	436 (30.4)	
Unknown	135 (9.4)	121 (8.4)	107 (7.5)	113 (7.9)	
Multimorbidities^c^					
None	244 (17.1)	257 (18.1)	263 (18.4)	312 (21.9)	<.001
1 of 11	460 (32.2)	491 (34.5)	524 (36.6)	527 (36.9)	
2 of 11	392 (27.4)	400 (28.1)	382 (26.7)	364 (25.5)	
≥3 of 11	335 (23.4)	274 (19.3)	261 (18.3)	225 (15.8)	
Self-rated health					
Excellent or very good	730 (51.0)	730 (51.1)	793 (55.5)	815 (57.1)	.003
Good	573 (40.0)	590 (41.3)	538 (37.6)	522 (36.6)	
Fair or poor	129 (9.0)	108 (7.6)	99 (6.9)	91 (6.4)	
SPPB score					
Low function (0-9)	845 (70.8)	801 (64.6)	769 (61.4)	726 (57.4)	<.001
High function (10-12)	349 (29.2)	439 (35.4)	484 (38.6)	539 (42.6)	
MVPA, mean (SD), min/d^a^	36.1 (26.1)	47.6 (29.8)	57.7 (33.9)	66.8 (35.7)	<.001

Abbreviations: BMI, body mass index (calculated as weight in kilograms divided by height in meters squared); MVPA, moderate-to-vigorous-intensity physical activity; NA, not applicable; OPACH, Objective Physical Activity and Cardiovascular Health; SPPB, short physical performance battery; WHI, Women’s Health Initiative.

^a^ Adjusted for awake wear time using the residuals method.

^b^ Quartile 1 indicates 0.6 to 4.0 or fewer hours per day of light physical activity; quartile 2, more than 4.0 to 4.8 or fewer hours per day; quartile 3, more than 4.8 to 5.6 or fewer hours per day; quartile 4, more than 5.6 to 10.4 hours per day.

^c^ Sum of cancer, cerebrovascular disease, cognitive impairment, sensory impairment, cardiovascular disease, chronic obstructive pulmonary disease, diabetes, frequent falls, urinary incontinence, depression, and osteoarthritis.

During up to 6.0 years of follow-up (median, 4.6 years; range, 0-6 years), there were 1277 cases of incident mobility disability over 22 353 person-years and 859 cases of persistent mobility disability over 21 449 person-years. Risk of incident mobility disability was lower across increasing quartiles of LPA (Table 2): HRs were strongest and approximately equivalent in the upper 2 quartiles (in model 1: HR, 0.54 [95% CI, 0.46-0.63] in Q4 vs 0.56 [95% CI, 0.48-0.65] in Q3), suggesting a possible threshold of the association between LPA and mobility disability. The minimally adjusted model (model 1) showed a beneficial association starting in Q2 of LPA with a 24% lower risk (HR, 0.76; 95% CI, 0.66-0.87) of incident mobility disability compared with Q1. Women in Q3 and Q4 of LPA exhibited even stronger beneficial associations, with 44% (HR, 0.56; 95% CI, 0.48-0.65) and 46% (HR, 0.54; 95% CI, 0.46-0.63) lower risks of incident mobility disability, respectively, compared with Q1 (*P* < .001 for trend). Further adjustment for confounders (model 2) resulted in a small attenuation, but the beneficial associations persisted (compared with Q1, HR: 0.78 [95% CI, 0.67-0.90] in Q2, 0.60 [95% CI, 0.51-0.71] in Q3, and 0.60 [95% CI, 0.51-0.71] in Q4; *P* < .001 for trend). Associations of LPA with incident mobility disability also persisted after additional adjustment for MVPA in model 3 with some attenuation (compared with Q1, HR: 0.87 [95% CI, 0.75-1.01] in Q2, 0.73 [95% CI, 0.62-0.85] in Q3, and 0.79 [95% CI, 0.67-0.93] in Q4; *P* < .001).

**Table 2.** Hazard Ratios for Light-Intensity Physical Activity and Incident Mobility Disability, Women’s Health Initiative Objective Physical Activity and Cardiovascular Health Study

Outcome	HR (95% CI) by quartile of physical activity^a^				*P* value^b^
	1	2	3	4	
**Incident mobility disability**^c^					
Incident events, rate^d^	84.3	62.5	44.2	41.1	NA
Model 1	1 [Reference]	0.76 (0.66-0.87)	0.56 (0.48-0.65)	0.54 (0.46-0.63)	<.001
Model 2	1 [Reference]	0.78 (0.67-0.90)	0.60 (0.51-0.71)	0.60 (0.51-0.71)	
Model 3	1 [Reference]	0.87 (0.75-1.01)	0.73 (0.62-0.85)	0.79 (0.67-0.93)	
**Persistent incident mobility disability**^e^					
Incident events, rate^d^	62.0	41.0	28.6	25.1	NA
Model 1	1 [Reference]	0.68 (0.58-0.81)	0.50 (0.42-0.61)	0.46 (0.38-0.56)	<.001
Model 2	1 [Reference]	0.72 (0.60-0.85)	0.55 (0.46-0.67)	0.52 (0.42-0.63)	
Model 3	1 [Reference]	0.82 (0.69-0.98)	0.70 (0.57-0.85)	0.73 (0.59-0.90)	

Abbreviations: HR, hazard ratio; NA, not applicable.

^a^ Quartile 1 indicates 0.6 to 4.0 or fewer hours per day of light physical activity; quartile 2, more than 4.0 to 4.8 or fewer hours per day; quartile 3, more than 4.8 to 5.6 or fewer hours per day; quartile 4, more than 5.6 to 10.4 hours per day.

^b^ Nonlinear light-intensity physical activity using cubic-spline method.

^c^ Model 1, adjusted for age, race/ethnicity, education (n = 5693); Model 2, model 1 with smoking, alcohol use, multimorbidities, self-rated health (n = 5654); Model 3, model 2 with moderate to vigorous–intensity physical activity (n = 5654).

^d^ Crude incidence rate per 1000 person-years.

^e^ Model 1, adjusted for age, race/ethnicity, education (n = 5278); Model 2, model 1 with smoking, alcohol use, multimorbidities, self-rated health (n = 5244); Model 3, model 2 with moderate to vigorous–intensity physical activity (n = 5244).

Similarly, significant associations were observed for LPA and persistent mobility disability (for Q4 in model 1, HR: 0.46 [95% CI, 0.38-0.56]; model 2, HR: 0.52 [95% CI, 0.42-0.63]; and model 3, HR: 0.73 [95% CI, 0.59-0.90]). Again, beneficial associations were observed beginning in Q2 and were stronger but of similar magnitude in Q3 and Q4 compared with Q1. In the fully adjusted model (model 3), women had an 18% (HR, 0.82; 95% CI, 0.69-0.98), 30% (HR, 0.70; 95% CI, 0.57-0.85), and 27% (HR, 0.73; 95% CI; 0.59-0.90; *P* < .001) lower risk of persistent mobility disability in Q2, Q3, and Q4, respectively, compared with Q1.

**Figure 1.**
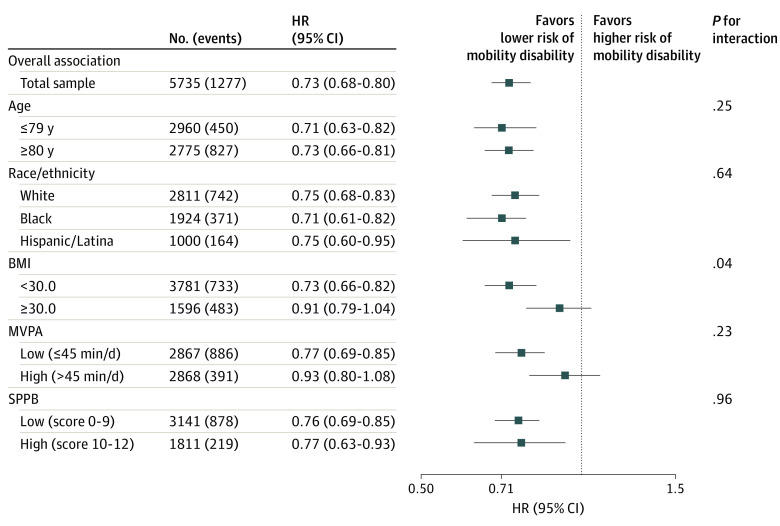
Stratified Models of Light-Intensity Physical Activity and Incident Mobility Disability BMI indicates body mass index (calculated as weight in kilograms divided by height in meters squared); HR, hazard ratio; MVPA, moderate-to-vigorous-intensity physical activity; and SPPB, Short Physical Performance Battery.

**Figure 2.**
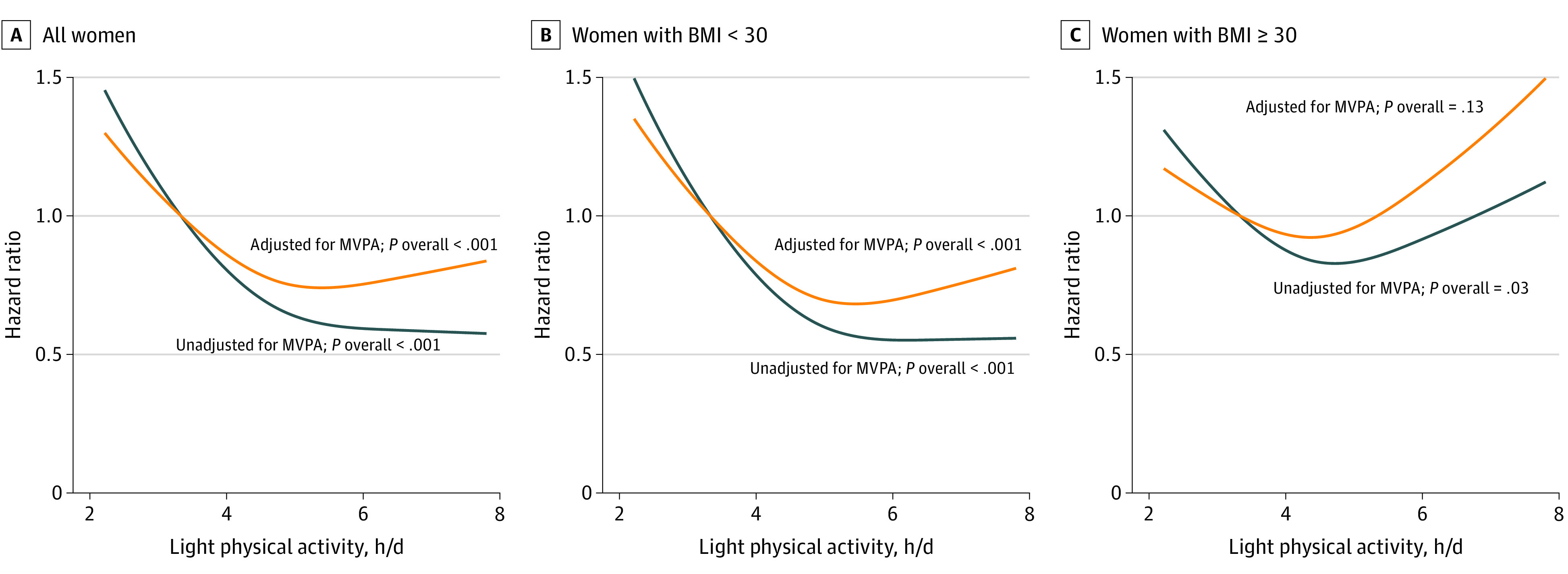
Stratified Dose-Response Models of Light Physical Activity and Incident Mobility Disability BMI indicates body mass index (calculated as weight in kilograms divided by height in meters squared) and MVPA, moderate-to-vigorous-intensity physical activity.

The multivariable-adjusted associations between LPA and incident mobility disability were consistent across strata of age (HR, 0.71 [95% CI, 0.63-0.82] for ≤79 years vs 0.73 [95% CI, 0.66-0.81] for >80 years; *P* = .25 for interaction), race/ethnicity (HR, 0.75 [95% CI, 0.68-0.83] for White participants, 0.71 [95% CI, 0.61-0.82] for Black participants, and 0.75 [95% CI, 0.60-0.95] for Hispanic/Latina participants; *P* = .64 for interaction), MVPA (HR, 0.77 [95% CI, 0.69-0.85] for low MVPA vs 0.93 [95% CI, 0.80-1.08] for high MVPA; *P* = .23 for interaction), and SPPB score (HR, 0.76 [95% CI, 0.69-0.85] for low SPPB score vs 0.77 [0.63-0.93] for high SPPB score; *P* = .96 for interaction) (Figure 1 and eFigure 2 in the Supplement). Although reduced risks of incident mobility disability were seen in women both with and without obesity, associations appeared stronger among women with a BMI less than 30.0 (HR, 0.73 [95% CI, 0.66-0.82] for a 1–interquartile range increment in LPA) compared with those with a BMI greater than or equal to 30.0 (HR, 0.91 [95% CI, 0.79-1.04]; *P* = .04 for interaction) (Figure 1). Although LPA was normally distributed across the population, among women with a BMI less than 30.0, after adjustment for demographic characteristics and confounders (model 2), greater LPA was associated with lower risk of incident mobility disability at 4, 5, and 6 hours of daily mean LPA compared with 3.3 hours (10th percentile of LPA: HR, 0.78 [95% CI, 0.72-0.85] for 4 hours per day; 0.60 [95% CI, 0.51-0.71] for 5 hours per day; and 0.55 [95% CI, 0.46-0.66] for 6 hours per day) (Figure 2 and eFigure 3 in the Supplement). Additional adjustment for MVPA again showed attenuation of the HRs, but a similar pattern was observed as in the models without MVPA adjustment.

To address possible residual confounding by poor health status, sensitivity analyses re-examined models for LPA and incident mobility disability after excluding women from the analytical sample who died within 1 year of follow-up, who reported fair or poor self-rated health, or who had 2 or more major chronic conditions. In no case were results meaningfully changed (eTable 1, eTable 2, and eTable 3 in the Supplement).

## Discussion

To our knowledge, this is the first study to examine the association of objectively measured LPA with incident mobility disability in a general population of older adults with intact mobility. Lower risk of incident mobility disability associated with increasing LPA was observed starting in the second lowest quartile of LPA. Women in the highest quartile of LPA had a 40% lower risk of incident mobility disability and a 48% lower risk of persistent mobility disability compared with women in the lowest quartile of LPA after adjustment for demographic and confounding factors. Women with a BMI less than 30.0 had a stronger beneficial association than those with a BMI greater than or equal to 30.0 kg/m^2^. Results suggest that high levels of LPA are not necessary to reduce the risk of mobility disability, because no further reductions were observed over approximately 5 hours of LPA per day. Overall, the interpretation of LPA and incident mobility disability was robust to consideration of MVPA as a possible confounder or effect modifier. The association of LPA with health outcomes was identified as a gap in current knowledge by the 2018 Physical Activity Guidelines Advisory Committee.^9^

To our knowledge, few studies have examined LPA and mobility among older adults.^25^ The LIFE Study reported a 15% lower risk of incident mobility disability associated with the addition of an increment of 30 minutes per day in LPA among highly sedentary adults aged 70 to 89 years with existing physical limitations.^26^ A study among community-dwelling adults aged 49 years or older with or at risk for knee osteoarthritis found a 42% lower risk of incident disability for the highest vs lowest quartile of objectively measured LPA, similar to the magnitude in our study.^27^ Although these studies are comparable in their use of accelerometer-measured LPA and focus on disability and health among older adults, our study is distinct in several ways. We focused on older women, who bear a disproportionate burden of mobility disability. Our examination of incident mobility disability among women with intact mobility at baseline supports LPA as potentially beneficial to all older women, not just those with existing physical limitations. Furthermore, because of our focus on mobility disability rather than poor health or a broader definition of physical function, our findings provide strong evidence for the translation of LPA into medical practice as a method for preserving mobility.

The beneficial association of LPA was stronger for women with lower BMI. Obesity in older women is strongly associated with poorer lower body function and walking ability.^28^ Within the WHI, obesity was associated with very high odds of incident mobility disability among women who survived to 85 years.^29^ Moreover, in the National Institutes of Health–AARP Diet and Health Study cohort and the Health, Aging and Body Composition study, beneficial associations between physical activity and mobility disability were weaker among adults with higher BMI levels.^30,31^ Taken together, these studies suggest that the potential favorable influence of PA on mobility disability may not overcome the increased risk related to higher BMI. However, older adults with excess weight can still potentially benefit from increased PA. In the Look AHEAD (Action for Health in Diabetes) trial, a nutritional and PA intervention among adults with excess weight and type 2 diabetes, individuals in the intervention arm experienced significant weight loss, improved physical fitness, and improved physical function.^32^

The physiological mechanisms that lead to mobility disability are not fully understood. In a systematic review and meta-analysis of activities of daily living among older adults, activities of daily living dependency was strongly associated with sarcopenia, low muscle strength, and low physical performance.^33^ Previous studies have found increased PA to be a protective factor against the development of sarcopenia among older adults.^34^ Additionally, trials among frail older adults have produced generally positive results of PA interventions with improvements in muscle strength, balance, and mobility, although the strength of these associations is inconsistent.^35^ Light-intensity PA is characterized by lower-required energy expenditure compared with MVPA but still requires engagement of muscle groups. Although few studies have been conducted on the physiological mechanisms associated with mobility disability and specifically LPA, positive associations have been found between LPA and muscle strength in the leg^36^ and bone mineral density,^37^ suggesting that LPA can provide similar physiological benefits as other forms of PA.

### Strengths and Limitations

The OPACH study included a large cohort of postmenopausal women from multiple sites, races/ethnicities, and socioeconomic backgrounds. This diversity increases the generalizability of our findings. This study also had minimal loss to follow-up, and the use of accelerometers allowed us to objectively measure LPA. We were able to capture PA through a reliable measure, and the cut points for intensity were based on a calibration study among this specific population.^21^ Although this study focused on older women, studies among men have found beneficial associations between LPA and all-cause mortality, suggesting the potential health benefits of LPA are likely not limited to women.^38^

This study also has some limitations. Mobility disability status was based on self-report, a method generally considered less reliable compared with objective measures. However, self-reported abilities to walk and climb stairs provide valuable information on the participants’ evaluation of their own physical limitations in usual life circumstances. Furthermore, our definition and collection method are comparable to those of the Behavioral Risk Factor Surveillance System, a telephone-based survey used for US statistics.^5^ Annual ascertainment of mobility is a potential limitation as shorter duration incident mobility disability with recovery in less than 1 year may not have been captured. Longer-term and persistent mobility disability is of greater interest given the effects on independence and mortality. Light-intensity PA was measured over a single 7-day baseline wear period, and we were unable to examine repeated measures. Nonetheless, the baseline measure most removed from the outcome reduces the possibility of reverse causation.

## Conclusions

Mobility is a vital component of quality of life and a hallmark of healthy aging. Identifying modifiable behaviors to reduce late-life mobility disability and its clinical and economic consequences is of huge public health importance, especially considering our aging US population. The results of this cohort study suggest that more time spent in LPA could have a notable influence on the health and well-being of postmenopausal women through maintaining mobility and therefore independence. The large pragmatic WHISH (WHI Strong and Healthy) trial,^39^ currently under way, will evaluate whether increasing LPA can prevent mobility disability. Our study findings suggest that all movement, not just MVPA, is important and that greater emphasis should be placed on promoting LPA for preserving mobility in later life.

## Notes

Supplement. **eFigure 1.** Strobe Diagram for Light Physical Activity Data in OPACH Study Followed for Incident Mobility Disability Through March 31, 2018 **eFigure 2.** Dose-Response Models of Light Physical Activity Incident Mobility Disability Stratified by Potential Effect Modifiers, WHI OPACH **eFigure 3.** Distribution of Light Physical Activity by Body Mass Index Status, WHI OPACH **eTable 1.** Hazard Ratios for Light Physical Activity and Incident Mobility Disability, WHI OPACH – Women With Incident Mobility Disability Events in First Year of Follow-up Excluded From Analytical Sample **eTable 2.** Hazard Ratios for Light Physical Activity and Incident Mobility Disability, WHI OPACH – Women With Self-Reported Fair or Poor Health Excluded From Analytical Sample **eTable 3.** Hazard Ratios for Light Physical Activity and Incident Mobility Disability, WHI OPACH – Women With 2 or More Chronic Conditions Excluded From Analytical Sample

## References

[1] US Census Bureau. Older people projected to outnumber children for first time in U.S. history. Accessed September 6, 2018. https://www.census.gov/newsroom/press-releases/2018/cb18-41-population-projections.html

[2] US Department of Health and Human Services. Child Health USA 2013. Health Resources and Services Administration; 2013.

[3] Centers for Disease Control and Prevention. The state of aging and health in America 2013. CDC; 2013.

[4] Spillman BC, Lubitz J The effect of longevity on spending for acute and long-term care. N Engl J Med. 2000;342(19):1409-1415. doi:10.1056/NEJM200005113421906 10805827

[5] Courtney-Long EA, Carroll DD, Zhang QC, Prevalence of disability and disability type among adults—United States, 2013. MMWR Morb Mortal Wkly Rep. 2015;64(29):777-783. doi:10.15585/mmwr.MM6429a2 26225475 PMC4584831

[6] Vos T, Barber R, Bell B, ; Global Burden of Disease Study 2013 Collaborators Global, regional, and national incidence, prevalence, and years lived with disability for 301 acute and chronic diseases and injuries in 188 countries, 1990-2013: a systematic analysis for the Global Burden of Disease Study 2013. Lancet. 2015;386(9995):743-800. doi:10.1016/S0140-6736(15)60692-4 26063472 PMC4561509

[7] Satariano WA, Guralnik JM, Jackson RJ, Marottoli RA, Phelan EA, Prohaska TR Mobility and aging: new directions for public health action. Am J Public Health. 2012;102(8):1508-1515. doi:10.2105/AJPH.2011.300631 22698013 PMC3464831

[8] Hardy SE, Kang Y, Studenski SA, Degenholtz HB Ability to walk 1/4 mile predicts subsequent disability, mortality, and health care costs. J Gen Intern Med. 2011;26(2):130-135. doi:10.1007/s11606-010-1543-2 20972641 PMC3019329

[9] Office of Disease Prevention and Health Promotion. 2018 physical activity guidelines advisory committee submits scientific report. March 5, 2018 Accessed February 28, 2019. https://health.gov/news-archive/blog-bayw/2018/03/2018-physical-activity-guidelines-advisory-committee-submits-scientific-report/

[10] Pahor M, Guralnik JM, Ambrosius WT, ; LIFE study investigators Effect of structured physical activity on prevention of major mobility disability in older adults: the LIFE study randomized clinical trial. JAMA. 2014;311(23):2387-2396. doi:10.1001/jama.2014.5616 24866862 PMC4266388

[11] LaCroix AZ, Bellettiere J, Rillamas-Sun E, ; Women’s Health Initiative (WHI) Association of light physical activity measured by accelerometry and incidence of coronary heart disease and cardiovascular disease in older women. JAMA Netw Open. 2019;2(3):e190419. doi:10.1001/jamanetworkopen.2019.0419 30874775 PMC6484645

[12] LaMonte MJ, Buchner DM, Rillamas-Sun E, Accelerometer-measured physical activity and mortality in women aged 63 to 99. J Am Geriatr Soc. 2018;66(5):886-894. doi:10.1111/jgs.15201 29143320 PMC5955801

[13] Carlson SA, Fulton JE, Schoenborn CA, Loustalot F Trend and prevalence estimates based on the 2008 Physical Activity Guidelines for Americans. Am J Prev Med. 2010;39(4):305-313. doi:10.1016/j.amepre.2010.06.006 20837280

[14] LaMonte MJ, Lee IM, Rillamas-Sun E, Comparison of questionnaire and device measures of physical activity and sedentary behavior in a multi-ethnic cohort of older women. JMPB. 2019;2(2):82-93. doi:10.1123/jmpb.2018-0057

[15] The Women’s Health Initiative Study Group Design of the Women’s Health Initiative clinical trial and observational study. Control Clin Trials. 1998;19(1):61-109. doi:10.1016/S0197-2456(97)00078-0 9492970

[16] Anderson GL, Manson J, Wallace R, Implementation of the Women’s Health Initiative study design. Ann Epidemiol. 2003;13(9)(suppl):S5-S17. doi:10.1016/S1047-2797(03)00043-7 14575938

[17] LaCroix AZ, Rillamas-Sun E, Buchner D, The Objective Physical Activity and Cardiovascular Disease Health in Older Women (OPACH) Study. BMC Public Health. 2017;17(1):192. doi:10.1186/s12889-017-4065-6 28193194 PMC5307783

[18] LaCroix AZ, Guralnik JM, Berkman LF, Wallace RB, Satterfield S Maintaining mobility in late life. II. Smoking, alcohol consumption, physical activity, and body mass index. Am J Epidemiol. 1993;137(8):858-869. doi:10.1093/oxfordjournals.aje.a116747 8484377

[19] Khokhar SR, Stern Y, Bell K, Persistent mobility deficit in the absence of deficits in activities of daily living: a risk factor for mortality. J Am Geriatr Soc. 2001;49(11):1539-1543. doi:10.1046/j.1532-5415.2001.4911251.x 11890596

[20] Willett W, Stampfer MJ Total energy intake: implications for epidemiologic analyses. Am J Epidemiol. 1986;124(1):17-27. doi:10.1093/oxfordjournals.aje.a114366 3521261

[21] Evenson KR, Wen F, Herring AH, Calibrating physical activity intensity for hip-worn accelerometry in women age 60 to 91 years: the Women’s Health Initiative OPACH Calibration Study. Prev Med Rep. 2015;2(2):750-756. doi:10.1016/j.pmedr.2015.08.021 26527313 PMC4625400

[22] Migueles JH, Cadenas-Sanchez C, Ekelund U, Accelerometer data collection and processing criteria to assess physical activity and other outcomes: a systematic review and practical considerations. Sports Med. 2017;47(9):1821-1845. doi:10.1007/s40279-017-0716-0 28303543 PMC6231536

[23] Guralnik JM, Simonsick EM, Ferrucci L, A short physical performance battery assessing lower extremity function: association with self-reported disability and prediction of mortality and nursing home admission. J Gerontol. 1994;49(2):M85-M94. doi:10.1093/geronj/49.2.M85 8126356

[24] Choi L, Liu Z, Matthews CE, Buchowski MS Validation of accelerometer wear and nonwear time classification algorithm. Med Sci Sports Exerc. 2011;43(2):357-364. doi:10.1249/MSS.0b013e3181ed61a3 20581716 PMC3184184

[25] Bauman A, Merom D, Bull FC, Buchner DM, Fiatarone Singh MA Updating the evidence for physical activity: summative reviews of the epidemiological evidence, prevalence, and interventions to promote “active aging”. Gerontologist. 2016;56(suppl 2):S268-S280. doi:10.1093/geront/gnw031 26994266

[26] Mankowski RT, Anton SD, Axtell R, ; LIFE Research Group Device-measured physical activity as a predictor of disability in mobility-limited older adults. J Am Geriatr Soc. 2017;65(10):2251-2256. doi:10.1111/jgs.15037 28799216 PMC5657432

[27] Dunlop DD, Song J, Semanik PA, Relation of physical activity time to incident disability in community dwelling adults with or at risk of knee arthritis: prospective cohort study. BMJ. 2014;348:g2472. doi:10.1136/bmj.g2472 24782514 PMC4004786

[28] Vincent HK, Vincent KR, Lamb KM Obesity and mobility disability in the older adult. Obes Rev. 2010;11(8):568-579. doi:10.1111/j.1467-789X.2009.00703.x 20059707

[29] Rillamas-Sun E, LaCroix AZ, Waring ME, Obesity and late-age survival without major disease or disability in older women. JAMA Intern Med. 2014;174(1):98-106. doi:10.1001/jamainternmed.2013.12051 24217806 PMC3963496

[30] DiPietro L, Jin Y, Talegawkar S, Matthews CE The joint associations of weight status and physical activity with mobility disability: The NIH-AARP Diet and Health Study. Int J Obes (Lond). 2019;43(9):1830-1838. doi:10.1038/s41366-018-0294-8 30575803 PMC8491301

[31] Koster A, Penninx BW, Newman AB, Lifestyle factors and incident mobility limitation in obese and non-obese older adults. Obesity (Silver Spring). 2007;15(12):3122-3132. doi:10.1038/oby.2007.372 18198323

[32] Foy CG, Lewis CE, Hairston KG, ; Look AHEAD Research Group Intensive lifestyle intervention improves physical function among obese adults with knee pain: findings from the Look AHEAD trial. Obesity (Silver Spring). 2011;19(1):83-93. doi:10.1038/oby.2010.120 20559303 PMC3408003

[33] Wang DXM, Yao J, Zirek Y, Reijnierse EM, Maier AB Muscle mass, strength, and physical performance predicting activities of daily living: a meta-analysis. J Cachexia Sarcopenia Muscle. 2020;11(1):3-25. doi:10.1002/jcsm.12502 31788969 PMC7015244

[34] Steffl M, Bohannon RW, Sontakova L, Tufano JJ, Shiells K, Holmerova I Relationship between sarcopenia and physical activity in older people: a systematic review and meta-analysis. Clin Interv Aging. 2017;12:835-845. doi:10.2147/CIA.S132940 28553092 PMC5441519

[35] de Labra C, Guimaraes-Pinheiro C, Maseda A, Lorenzo T, Millán-Calenti JC Effects of physical exercise interventions in frail older adults: a systematic review of randomized controlled trials. BMC Geriatr. 2015;15:154. doi:10.1186/s12877-015-0155-4 26626157 PMC4667405

[36] Chahal J, Lee R, Luo J Loading dose of physical activity is related to muscle strength and bone density in middle-aged women. Bone. 2014;67:41-45. doi:10.1016/j.bone.2014.06.029 24999224

[37] Chastin SFM, Mandrichenko O, Helbostadt JL, Skelton DA Associations between objectively-measured sedentary behaviour and physical activity with bone mineral density in adults and older adults, the NHANES study. Bone. 2014;64:254-262. doi:10.1016/j.bone.2014.04.009 24735973

[38] Jefferis BJ, Parsons TJ, Sartini C, Objectively measured physical activity, sedentary behaviour and all-cause mortality in older men: does volume of activity matter more than pattern of accumulation? Br J Sports Med. 2019;53(16):1013-1020. doi:10.1136/bjsports-2017-098733 29440040 PMC6691867

[39] Women’s Health Initiative strong and healthy study (WHISH). ClinicalTrials.gov identifier: NCT02425345. Updated October 5, 2020 Accessed December 27, 2020. https://clinicaltrials.gov/ct2/show/NCT02425345

