# Supplementary material for: Evaluation of Light Physical Activity Measured by Accelerometry and Mobility Disability During a 6-Year Follow-up in Older Women
Source: JAMA Netw Open. 2021 Feb 23;4(2):e210005. doi: 10.1001/jamanetworkopen.2021.0005 (PMC7903251; doi:10.1001/jamanetworkopen.2021.0005)
Supplement: Supplement. — eFigure 1. Strobe Diagram for Light Physical Activity Data in OPACH Study Followed for Incident Mobility Disability Through March 31, 2018 eFigure 2. Dose-Response Models of Light Physical Activity Incident Mobility Disability Stratified by Potential Effect Modifiers, WHI OPACH eFigure 3. Distribution of Light Physical Activity by Body Mass Index Status, WHI OPACH eTable 1. Hazard Ratios for Light Physical Activity and Incident Mobility Disability, WHI OPACH – Women With Incident Mobility Disability Events in First Year of Follow-up Excluded From Analytical Sample eTable 2. Hazard Ratios for Light Physical Activity and Incident Mobility Disability, WHI OPACH – Women With Self-Reported Fair or Poor Health Excluded From Analytical Sample eTable 3. Hazard Ratios for Light Physical Activity and Incident Mobility Disability, WHI OPACH – Women With 2 or More Chronic Conditions Excluded From Analytical Sample [file jamanetwopen-e210005-s001.pdf]

# Supplementary Online Content

Glass NL, Bellettiere J, Jain P, LaMonte MJ, LaCroix AZ; Women's Health Initiative. Evaluation of light physical activity measured by accelerometry and mobility disability during a 6-year follow-up in older women. *JAMA Netw Open*. 2021;4(2):e210005. doi:10.1001/jamanetworkopen.2021.0005

**eFigure 1.** Strobe Diagram for Light Physical Activity Data in OPACH Study Followed for Incident Mobility Disability Through March 31, 2018
**eFigure 2.** Dose-Response Models of Light Physical Activity Incident Mobility Disability Stratified by Potential Effect Modifiers, WHI OPACH
**eFigure 3.** Distribution of Light Physical Activity by Body Mass Index Status, WHI OPACH
**eTable 1.** Hazard Ratios for Light Physical Activity and Incident Mobility Disability, WHI OPACH – Women With Incident Mobility Disability Events in First Year of Follow-up Excluded From Analytical Sample
**eTable 2.** Hazard Ratios for Light Physical Activity and Incident Mobility Disability, WHI OPACH – Women With Self-Reported Fair or Poor Health Excluded From Analytical Sample
**eTable 3.** Hazard Ratios for Light Physical Activity and Incident Mobility Disability, WHI OPACH – Women With 2 or More Chronic Conditions Excluded From Analytical Sample

This supplementary material has been provided by the authors to give readers additional information about their work.

© 2021 Glass NL et al. *JAMA Network Open.*

**eFigure 1.** Strobe Diagram for Light Physical Activity Data in WHI, OPACH Study Followed for Incident Mobility Disability Through March 31, 2018

Enrolled in OPACH Study, 2012-2014
n=7058

n=10 excluded, death prior to receipt of accelerometer
n=327 excluded, did not return accelerometer
n=232 excluded, returned accelerometer with no usable data
n=107 excluded, did not have at least 1 day with ≥ 10

Women with physical activity data
n=6382

n=602 excluded, existing mobility disability at baseline

Women with intact mobility at baseline
n=5780

n=45 excluded, no follow-up mobility data after OPACH

**Data Available for Primary Analysis of All Incident Mobility Disability**
**n=5735**

22,353 Person-years of follow-up
Incident Mobility Disability, n=1277

n=418 excluded, incident mobility disability with reported recovery during follow-up period

**Data Available for Secondary Analysis of Only Persistent Incident Mobility Disability**
**n=5317**

21,449 Person-years of follow-up

© 2021 Glass NL et al. *JAMA Network Open.*

**eFigure 2.** Dose-Response Models of Light Physical Activity Incident Mobility Disability Stratified by Potential Effect Modifiers, WHI OPACH

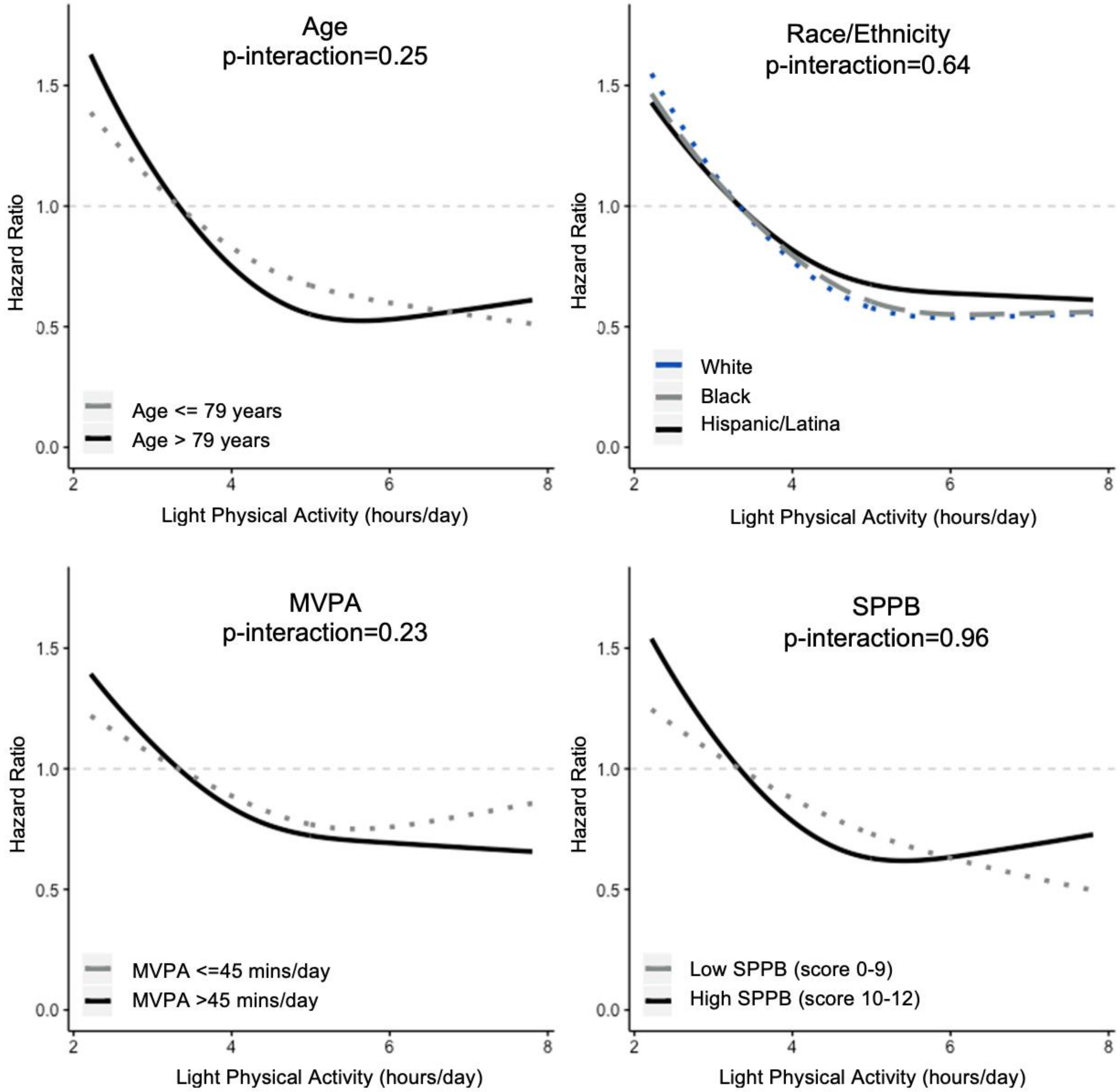


Abbreviations: MVPA (moderate-to-vigorous physical activity), SPPB (short performance physical battery)
Models adjusted for age, race/ethnicity, education, smoking, alcohol use, multi-morbidity, and self-rated health (variable excluded when used for stratification)

© 2021 Glass NL et al. *JAMA Network Open.*

**eFigure 3.** Distribution of Light Physical Activity by Body Mass Index Status, WHI OPACH

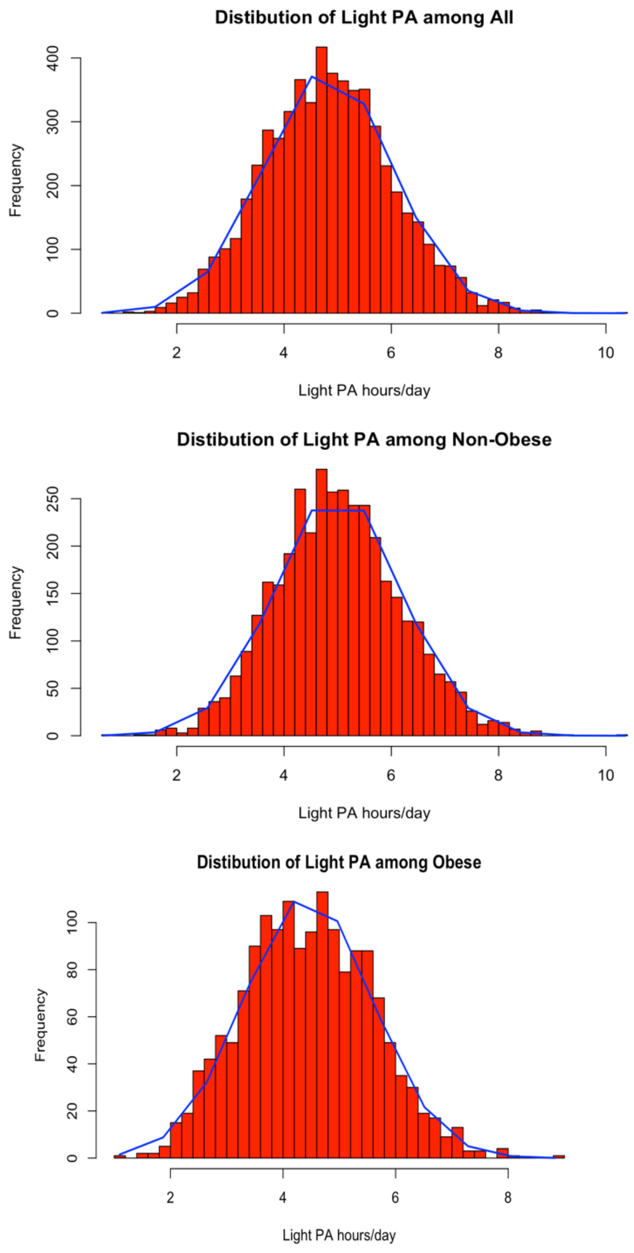


© 2021 Glass NL et al. *JAMA Network Open.*

**eTable 1.** Hazard Ratios for Light Physical Activity and Incident Mobility Disability, WHI OPACH – Women With Incident Mobility Disability Events in First Year of Follow-up Excluded From Analytical Sample

| | **Time spent in light physical activity (quartile)** | | | |
|---|---|---|---|---|
| | **Q1** | **Q2** | **Q3** | **Q4** |
| | **0.6-4.0 hours/day** | **4.0-4.8 hours/day** | **4.8-5.6 hours/day** | **5.6-10.4 hours/day** |
| | **n=1299** | **n=1338** | **n=1367** | **n=1374** |
| **Outcome** | **HR (95% CI)** | **HR (95% CI)** | **HR (95% CI)** | **HR (95% CI)** |
| Incident mobility disability [a] | | | | |
| Model 1 | 1.00 (ref.) | 0.76 (0.64-0.89) | 0.58 (0.49-0.69) | 0.54 (0.46-0.65) |
| Model 2 | 1.00 (ref.) | 0.78 (0.66-0.91) | 0.62 (0.52-0.74) | 0.60 (0.50-0.72) |
| Model 3 | 1.00 (ref.) | 0.86 (0.73-1.02) | 0.74 (0.62-0.89) | 0.77 (0.64-0.94) |

Abbreviation: Q1, first quartile; Q2, second quartile; Q3, third quartile; Q4, fourth quartile; HR, hazard ratio.

[a] Model 1: adjusted for age, race/ethnicity, education (n=5342; n-events=1003) ; Model 2: model 1 + smoking, alcohol use, comorbidity, self-rated health (n=5307; n-events=997); Model 3: model 2 + MVPA (n=5307; n-events=997)

© 2021 Glass NL et al. *JAMA Network Open.*

**eTable 2.** Hazard Ratios for Light Physical Activity and Incident Mobility Disability, WHI OPACH – Women With Self-Reported Fair or Poor Health Excluded From Analytical Sample

| | **Time spent in light physical activity (quartile)** | | | |
|---|---|---|---|---|
| | **Q1** | **Q2** | **Q3** | **Q4** |
| | **0.6-4.0 hours/day** | **4.0-4.8 hours/day** | **4.8-5.6 hours/day** | **5.6-10.4 hours/day** |
| | **n=1303** | **n=1320** | **n=1331** | **n=1337** |
| **Outcome** | **HR (95% CI)** | **HR (95% CI)** | **HR (95% CI)** | **HR (95% CI)** |
| Incident Mobility Disability [a] | | | | |
| Model 1 | 1.00 (ref.) | 0.74 (0.64-0.87) | 0.57 (0.48-0.67) | 0.56 (0.47-0.67) |
| Model 2 | 1.00 (ref.) | 0.77 (0.66-0.90) | 0.60 (0.50-0.71) | 0.62 (0.52-0.74) |
| Model 3 | 1.00 (ref.) | 0.86 (0.73-1.01) | 0.72 (0.60-0.85) | 0.80 (0.67-0.96) |

Abbreviation: Q1, first quartile; Q2, second quartile; Q3, third quartile; Q4, fourth quartile; HR, hazard ratio.

[a] Model 1: adjusted for age, race/ethnicity, education (n=5256; n-events=1063) ; Model 2: model 1 + smoking, alcohol use, comorbidity, self-rated health (n=5239; n-events=1061); Model 3: model 2 + MVPA (n=5239; n-events=1061)

© 2021 Glass NL et al. *JAMA Network Open.*

**eTable 3.** Hazard Ratios for Light Physical Activity and Incident Mobility Disability, WHI OPACH – Women With 2 or More Chronic Conditions Excluded From Analytical Sample

| | **Time spent in light physical (quartile)** | | | |
|---|---|---|---|---|
| | **Q1** | **Q2** | **Q3** | **Q4** |
| | **1.1-4.1 hours/day** | **4.1-4.9 hours/day** | **4.9-5.7 hours/day** | **5.7-10.4 hours/day** |
| | **n=704** | **n=748** | **n=787** | **n=839** |
| **Outcome** | **HR (95% CI)** | **HR (95% CI)** | **HR (95% CI)** | **HR (95% CI)** |
| Incident Mobility Disability [a] | | | | |
| Model 1 | 1.00 (ref.) | 0.70 (0.55-0.88) | 0.58 (0.45-0.74) | 0.55 (0.43-0.70) |
| Model 2 | 1.00 (ref.) | 0.67 (0.53-0.85) | 0.59 (0.46-0.76) | 0.55 (0.43-0.71) |
| Model 3 | 1.00 (ref.) | 0.75 (0.59-0.96) | 0.72 (0.56-0.93) | 0.75 (0.58-0.98) |

Abbreviation: Q1, first quartile; Q2, second quartile; Q3, third quartile; Q4, fourth quartile; HR, hazard ratio.

[a] Model 1: adjusted for age, race/ethnicity, education (n=3056; n-events=500) ; Model 2: model 1 + smoking, alcohol use, comorbidity, self-rated health (n=3047; n-events=500); Model 3: model 2 + MVPA (n=3047; n-events=500)

© 2021 Glass NL et al. *JAMA Network Open.*
